# Impact of Reproductive Health Education Seminars on College Students’ Contraception and Safe Sex Knowledge and Behaviors

**DOI:** 10.3390/pharmacy13020039

**Published:** 2025-03-03

**Authors:** Marisa Marcath, Kayla Craig, Mary Beth O’Connell

**Affiliations:** 1 Doctor of Pharmacy Program, Eugene Applebaum College of Pharmacy and Health Sciences, Wayne State University, Detroit, MI 48201, USA; mmarcath@wayne.edu (M.M.); gw4209@wayne.edu (K.C.); 2 Pharmacy Practice Department, Eugene Applebaum College of Pharmacy and Health Sciences, Wayne State University, Detroit, MI 48201, USA

**Keywords:** unintended pregnancies, reproductive health, contraception, college students, health education, learning, pharmacy, student pharmacists

## Abstract

In the United States, 41.6% of all pregnancies are unintended. This disproportionately affects women 20 to 24 years old. The high rate of unintended pregnancy among college students is associated with a lack of or incomplete formal education on reproductive health in grade and high school. The purpose of this study was to evaluate the outcomes of health education seminars designed to reduce reproductive health knowledge gaps among college-aged students and increase their safe sex behavior (health protection/prevention). Student pharmacists offered eighteen one-hour health education programs on fertility, contraception, and emergency contraception to college student attendees via live and Zoom-based presentations. Pre- and post-program quizzes, a post-program performance evaluation, and a post-program behavior change survey were completed anonymously. The response rate was 94.8% (*n* = 153 attendees). Post-program quiz scores (84%) were significantly higher than pre-program quiz scores (56%, *p* ≤ 0.001). The greatest increases in knowledge were about sperm survival, correct condom use, and fertility windows. The two-month follow-up survey responses revealed more confidence with birth control decisions, increased awareness of emergency contraception items, increased safe sex behaviors, and increased condom usage. Students reported that the seminars were helpful for preventing future unintended pregnancies. Reproductive health knowledge gaps can be minimized, and some self-reported safe sex behaviors can be improved with health education programs implemented at a university.

## 1. Introduction

In the United States (U.S.), 41.6% of all pregnancies in 2019 were unintended [1]. The U.S. Department of Health and Human Services declared unintended pregnancies a public health epidemic, and they have been included in the Healthy People 2030 public health initiatives [2]. The global unintended pregnancy rate for 2015–2019 was 48% [3]. This was highest in Latin America (68%) and lowest in sub-Saharan Africa (42%). The rate for other regions was 53% in East and Southeast Asia, 52% in West Asia and North Africa, 47% in Oceania, 46% in Europe and North America combined, and 44% in Central and South Asia. The percentages of pregnancies that were unintended varied by the country’s income, i.e., high, 47%; middle, 49%; and low, 43%. Unintended pregnancies are associated with negative outcomes for not only the woman but the infant as well [2,3,4,5]. Women who experienced an unintended pregnancy were more likely to delay their prenatal care, experienced violence, and developed mental health problems. In turn, these infants had higher risk for preterm birth and mental and physical health problems, which could make them more likely to struggle in school. Preventing these unintended pregnancies could potentially improve women’s, infants’, and children’s health.

The unintended pregnancy rate in the U.S. disproportionately affected women who were between 18 and 24 years old [6], had low income [6], never completed high school [6], were non-Hispanic Black or African American [1,6] or Hispanic [1], or were unmarried [1]. The unintended pregnancy rate of 51.1% for 20- to 24-year-olds was higher than the national average of 41.6% [1]. In college students, students with unintended pregnancies had a 65% higher college dropout rate than those without an unintended pregnancy [7]. The high rate of unintended pregnancy among college students has been associated with a lack of complete formal education on reproductive health in middle or high school [8].

Reproductive health literacy gaps exist among college-aged women and men. Less than 55% of college students knew that a woman can become pregnant during her period and that sperm can live in a woman’s body for 5 to 7 days [9]. In a large national survey of single adults in the U.S. aged 18–29 years old, 66% of the sample strongly agreed and 24% somewhat agreed that “I have all the information I need to avoid an unplanned pregnancy.” Despite this overconfidence in their fertility, sexuality, and contraceptive knowledge, 78% of men and 45% of women stated that they know little or nothing about birth control pills [8]. Forty percent of respondents agreed with the statement “It doesn’t matter whether you use birth control or not, when it’s your time to get pregnant it will happen,” and over forty percent believed the chance of becoming pregnant while using birth control as contraception within a year of using it was 50/50 [8], despite it being 93% effective with regular use [10]. These data highlight the gaps in grade and high school sex and reproductive health programs and the need for reproductive health education in college-aged students, who are disproportionately affected by unintended pregnancies.

College can be a period during which students engage in their first sexual activity. A study in China sampled 10,164 sexually active students across 49 universities to examine the relationship between the age of first sexual intercourse (AFSI) and knowledge, attitudes, and practices (KAP) regarding reproductive health and unplanned pregnancy [11]. The investigators found the AFSI in Chinese students occurred during their college years, and individuals with a younger AFSI have a higher risk of an unplanned pregnancy. In the U.S., about 20% of adolescents have had sex by 15 years old, which increases to about 78% by the age of 20 [12]. These results strengthen the argument that reproductive health education should be more present in colleges and, even earlier, in high school.

Since 1980, the U.S. Office of Disease Prevention and Health Promotion (CDC) has created national objectives for subsequent decades to improve the health and well-being of Americans and guide public health efforts. Decreasing the rate of unintended pregnancies in high-risk populations is a Healthy People 2030 public health initiative with various objectives [5]. Some objectives emphasize that interventions to increase birth control use are critical for the prevention of unintended pregnancies. Some interventions have been made to increase access to contraceptives and health care providers’ ability to counsel these patients on contraception. For example, the implementation of pharmacist-prescribed birth control in community pharmacies, which is practiced in 30 states and the District of Columbia [13], has been estimated to decrease unintended pregnancies by 51 for Medicaid recipients and save the state of Oregon USD 1.6 million a year [14].

The World Health Organization (WHO) in its Sustainable Development Goals for 2030 also focuses on decreasing the rates of unintended pregnancies [4]. Some of the goals of Target 3.7 are to increase access to reproductive and sexual health services, including access to family planning and safe and effective contraception. Recently, the WHO became active in increasing the number of pharmacies offering safe, effective, and accessible contraception [15].

According to the health belief model, people who understand the likelihood [susceptibility] and consequences [severity] of an unintended pregnancy and believe they can implement preventive measures [self-efficacy] to decrease the likelihood of an unintended pregnancy [benefits] are more likely to change their behaviors, especially if deterrents [barriers] are removed [16]. Thus, within the world, increasing education and health services to prevent unintended pregnancies, empowering women and trans people with knowledge and contraception, and increasing health care services are needed. The primary purposes of this “cues to act” [16] health education program were to quantify the reproductive health knowledge gaps among college-aged students in a U.S. urban university setting and determine their learning and behavior changes after an interactive seminar devoted to fertility, sexuality, contraception, and contraception services. A secondary purpose was to evaluate the seminars’ impact on reproductive health advocacy.

## 2. Materials and Methods

Our study had a pre/post-program survey design with a follow-up survey conducted two months after seminars delivered to college students at Wayne State University in Detroit, Michigan, U.S.

### 2.1. Student Pharmacist Training

Forty-four second-, third-, and fourth-year student pharmacists participated in an hour-long #PlanA training session. Student pharmacists reviewed their contraception lecture notes (4 h of material) from the endocrinology pharmacotherapy module before the training. All student pharmacists had the same level of background knowledge on this topic from the curriculum. Training provided the student pharmacists with detailed information on the slide set, how to conduct the seminars, presentation skills, and study details. At the beginning and conclusion of this training, student pharmacists completed 21-question pre- and post-training tests to document their proficiency with the material. These were the same questions seminar attendees and trivia participants (for a different project) would be answering. Students needed to score above 90% to deliver a seminar. If the score was less than that, student pharmacists (*n* = 2) re-reviewed the materials and performed a retake test until achieving success.

### 2.2. Presentation

Cognitive constructionism theory [17] and the rational model [16] were used to create the seminar. Learners would build on past knowledge with active learning to explore the various contraception options that would be best for them, learn new behaviors in order to have safer sex, and learn how to advocate for their reproductive health (cognitive constructionism) [17]. The rational model states that by increasing their knowledge, the learner will use this knowledge to change their attitudes and behaviors [16]. The learning objectives for this program were as follows:

- Compare and contrast hormonal contraceptives;
- Describe when women and transmen are most fertile;
- Select the contraception therapies with the greatest efficacy;
- Explain how to use a male condom;
- Know how to use and access emergency contraception;
- Advocate for the greater availability of contraception at lower costs.

The 75-slide presentation included information on anatomy; menstrual cycle physiology; fertility; hormonal, nonhormonal, and emergency contraception; and contraception costs and access. The presentation was titled “#PlanA: Through Education and Advocacy Empower Students to Practice Safe Sex Behaviors”. “#PlanA” was intended to draw in a college audience with the use of a hashtag, with PlanA being a play on words of the emergency contraceptive levonorgestrel, which has the well-known brand name Plan B in the U.S. The title was meant to insinuate that if you educate yourself on safe sex behaviors, which is Plan A, you will not need to use Plan B, the emergency contraceptive levonorgestrel. The research aspects of this project were included in the presentation. Four multiple-choice knowledge checkpoint questions about major topics were asked to ensure attendees understood the critical information and to create active participation.

The reproductive health advocacy issues chosen were increasing the access, availability, and cost of the university health clinic’s emergency contraception and supporting the Michigan House of Representatives’ proposed bills for Michigan pharmacist-prescribed birth control in community pharmacies. Each advocacy issue was included as an embedded video. One video was provided by the university’s Students for Reproductive Justice organization, which discussed efforts to expand and decrease the cost of emergency contraception on campus. The second video was provided by Michigan Representatives Kara Hope and Stephanie Young. The legislators described the importance of pharmacist-prescribed birth control due to a lack of access to contraception, with pharmacists being one of the most accessible health care providers, and the advocacy communications required with all state legislators to ask for their support of House Bills 5436 (pharmacy service) [18] and 5435 (service funding) [19] to assist with the bills’ passage.

### 2.3. Student Attendee Recruitment

All admitted undergraduate, graduate, and post-doctoral university students could attend a seminar. Student recruitment occurred from January to August 2024. Seminar promotion occurred primarily through digital media. Flyers included information on the benefits of attendance, a goody bag description, the mention of gift cards for survey completion, and a QR code to register. Electronic posters advertising our semester-long seminars were displayed on video screens in various campus buildings, included in campus newsletter emails, displayed on university websites, and posted in student center elevators and on social media (Instagram, Facebook, and Snapchat). Students received a reminder email a few days before their selected date. Incoming and first-year student pharmacists could receive a co-curricular pharmacy credit for attendance. We advertised our seminars and promoted attendance but were only able to collect data from students willing to attend. Thus, we had a convenience sample (Table 1) with which to evaluate our seminar’s impact.

### 2.4. Seminar

Eighteen hour-long seminars were delivered by trained student pharmacists, with at least one student investigator and faculty advisor present. Seminars were delivered at varying times of day (midday to evening) and days of the week in the student center, lecture rooms on the main and pharmacy and health sciences campuses, and two dormitories. Later presentations were also livestreamed on Zoom. We offered one to two seminars a week. Seminars were conducted even if the audience was small. The hour-long seminar covered all aspects of the education and advocacy components mentioned above. During the presentation, examples of hormonal and nonhormonal birth control products from the Birth Control Pharmacists’ toolkit [20] were passed around to the attendees. This kit contains various contraceptive items, with tags attached containing key information about the product, which are intended for hands-on educational purposes. Models of an intrauterine device (IUD) in a uterus and a Nexplanon^®^ skin implant in an arm were circulated. A demonstration of how to correctly put on and take off a condom was carried out with a plastic penis model.

Students were given a goody bag at the live presentations. The bag contained a critical take-away points sheet from the presentation, a QR code that led to a copy of the presentation slides, a basic reproductive anatomy diagram, a menstrual cycle figure, a Planned Parenthood hormonal contraceptive pamphlet, a pregnancy test, and two condoms.

### 2.5. Development of Pre- and Post-Program Quiz, Post-Seminar Program Evaluation, and 2-Month Follow-Up Survey

The quizzes, program evaluation, and the 2-month follow-up survey questions were created in Qualtrics (Provo, UT, U.S.). Pre- and post-program quizzes were designed to measure changes in the knowledge of the participants before and after the presentation. The program evaluation included standard investigator-developed questions about the program’s delivery, logistics, and impact. A 2-month time frame was chosen for the follow-up survey of behavior changes to give attendees enough time to consider contraceptive changes and implement them, choose safer sex behaviors, and email a letter of support for pharmacist-prescribed birth control to their representative if desired. The follow-up survey also needed to be done before the grant ended.

The pre- and post-program quizzes included ten knowledge-based multiple-choice questions that represented the key learning points, with one to two questions from each of the major areas of the seminar. Quiz questions and their answers are listed in Table 2. The knowledge quiz was piloted by six reviewers to ensure the questions were clear and accurate. The reviewers were three male student pharmacists, one female non-health care college student, one female teacher, and a male auto body technician. The pre-program quiz also included six demographic questions. The post-program quiz also included fourteen program evaluation and self-assessment questions (Table 3). The pre-program quiz, with demographic questions, and the post-program quiz, with program evaluation items, had a 5.9 and 7.0 Flesch–Kincaid grade level readability, respectively.

The follow-up survey assessed any changes in attitudes or behaviors regarding reproductive health and sexual behaviors as a result of seminar attendance and advocacy efforts. The 15-item follow-up survey (Table 4) used skip logic, so only students using birth control answered questions relevant to birth control, and only those students sexually active within the last two months answered questions about safe sex behavior changes. This survey had a 6.6 Flesch–Kincaid grade level readability.

### 2.6. Quiz, Program Evaluation, and Follow-Up Survey

Because of the sensitive nature of the answers, all students were given a unique five-digit code to keep responses anonymous. They completed the pre-program quiz and demographic questions immediately before the seminar and the post-program quiz and program evaluation questions immediately after the seminar using their specific code. Upon completion of the post-program quiz and program evaluation, participants would show to a student investigator their phone or computer screen showing their completion to receive a USD 5 gift card. This process was outlined at the beginning of the presentation.

### 2.7. Two-Month Follow-Up Survey

Two months after each seminar, the follow-up survey was emailed to the attendees. The email included the attendee’s original code and a link to the Qualtrics survey. After a week, another email was sent to nonresponders. A USD 10 gift card was emailed to attendees who completed a separate linked survey to capture their personal information.

### 2.8. Analysis

Only students that completed both the pre- and post-program quizzes were included in our analysis. Pre- and post-program quiz codes were matched, and data were analyzed using SPSS v29 software [21]. Due to the low numbers of transgender and nonbinary attendees, these attendees were excluded during gender subgroup statistical tests. Descriptive statistics were used for the demographics and frequencies from the quiz, program evaluation, and survey items. Total scores for the pre- and post-knowledge quiz items and the total amount learned were analyzed using a paired T-test for participants’ pre- and post-quizzes and using a nonpaired T-test for various demographics. The McNemar test was used for comparing the answers to each quiz question before and after the seminar. Mann–Whitney tests were used to compare demographic characteristics. The Chi-squared test was used for evaluating quiz performances across demographic variables for each item in the pre- and post-program quizzes, the program evaluation, and the follow-up survey and for comparisons between individual items. A *p* value of ≤0.05 was considered significant.

## 3. Results

### 3.1. Student Attendee Demographics 

In total, 153 students attended 1 of the 18 seminars; 147 in person and 6 by Zoom. Both surveys were completed by 145 students (a 94.8% response rate). Students were 22.5 ± 4.7 years old and predominantly White and female. Demographic data are provided in Table 1.

### 3.2. Pre- and Post-Program Quizzes

The pre- and post-program quiz results are shown in Table 2. The total post-program quiz knowledge scores were significantly higher than pre-program knowledge quiz scores (84% vs. 56%, respectively, *p* < 0.001). Nine of the ten content questions were significantly higher documenting knowledge improvement. A majority of attendees (90.0%) sent or planned to send an email in support of pharmacist-prescribed contraception to their legislator. Nearly all (99.3%) attendees felt it was important to continue offering #PlanA seminars to the Wayne State University student body.

Seminar evaluation data are shown in Table 3. Students agreed their knowledge about reproductive health and safe sex behaviors improved as a result of the seminar. They agreed the presentation was beneficial to help prevent an unintended pregnancy and that being able to obtain contraception from a pharmacist would help to decrease unintended pregnancies. Many attendees provided comments praising the program and its components. Some examples of these comments follow.

“It was a great seminar, with lots of important information! I like that the information that was shared was easily ‘digestible’”.

“Very informative, liked that they had models of the contraceptives. Very engaging with questions incorporated into the presentation”.

“The #PlanA seminar was very informative, and it was nice to be able to physically interact with the different forms of birth control as they were being discussed. The presenters also seemed to be very knowledgeable about the topic, making it easy to ask questions”.

“I enjoyed being able to attend this event to have these kinds of discussions and being able to be more educated due to the lack of real education in school systems”.

### 3.3. Follow-Up Survey

The follow-up survey results are shown in Table 4. Attendees felt that the seminars were helpful for preventing unintended pregnancies and made them more confident in making the correct birth control and safe sex behavior decisions. Following the presentation, 75.5% of attendees stated that they changed their sexual behaviors to become safer and prevent unintended pregnancies as a result of the seminars.

The survey also captured the number of attendees who emailed their representatives a letter to support pharmacists prescribing birth control. Of the students who had or were going to send a legislator an advocacy letter, 22.5% were student pharmacists. Eighty-five percent of student pharmacist attendees sent (*n* = 2) or were going to send a letter (*n* = 20).

### 3.4. Gender Differences

Data from the pre- and post-program quizzes showed that there were some differences between female and male attendees. The pre- and post-program quiz gender differences are shown in Table 5. Male attendees were older students, and female attendees were younger students. Undergraduate students were more likely to be female, and graduate and postdoc students were more likely to be male. Female attendees knew more than the men at baseline (*p* = 0.022); however, their post-quiz scores were similar (*p* = 0.220). The male scores increased by 0.7 more than the female scores, though the amount learned between genders was not significantly different (*p* = 0.200).

From the follow-up survey, about 73% of female and 60% of male attendees strongly agreed or agreed that their use of condoms to prevent an unintended pregnancy increased in response to their learning from the seminar. In terms of advocacy, 16.5% of female and 16.6% of male attendees stated ‘yes’ to having emailed their Michigan House of Representatives legislator a letter to support pharmacists prescribing birth control. Many attendees stated they would be sending a letter (68.4% of female and 50.0% of male attendees). Some attendees stated they would not be sending an advocacy letter (15.2% of female and 33.3% of male attendees).

### 3.5. Subgroup Differences

A subgroup analysis was conducted using the student attendee demographic responses. These results are in Table 5. On average, undergraduate students were younger and graduate students were older. Our analyses showed that younger students (18–22 years old) knew more at baseline than older students (23–46 years old), but this difference was not significant in the post-program quiz score. Health care students were more White, and non-health care students were more non-White. White students’ pre- and post-program quiz scores were significantly higher, but overall learning was not significantly different between the two groups.

## 4. Discussion

Per the health belief model, students need to understand the interventions that they can implement to achieve their health goals and identify ways to overcome barriers [16]. Health education programs can influence knowledge and behavior changes to improve health behaviors and outcomes [16,17]. Our students improved their knowledge about fertility, safe sex, contraception, emergency contraception, and the cost of contraception and their access to it, including that pharmacists can prescribe birth control. Students self-reported some positive changes in their sexual behaviors within the two months following the seminar. Most students found the seminars informational and helpful for preventing unintended pregnancies. Many students commented on the need for a program like this to be implemented at universities and expressed gratitude for the seminars that were held.

Some studies have quantified knowledge gaps and identified common themes in college students’ knowledge about reproductive health and their attitudes on the subject. The American Association of Community Colleges created a project [7] to integrate pregnancy prevention and planning into community college courses. Instructors chose whether they wanted to participate and could redesign the provided curricula or integrate a few additional activities into their course. Students exposed to the redesigned curriculum completed pre- and post-course surveys assessing their change in knowledge, attitudes, and behavioral intent. The survey questions were not knowledge-based, but self-evaluation statements on their beliefs and confidence regarding pregnancy and healthy relationships. The study found that students were more confident in their knowledge of unplanned pregnancies and birth control methods and were less likely to have unprotected sex. Although the students self-reported these positive changes, the survey did not ask any knowledge-based questions to determine their retention or understanding of the material like our surveys.

Gender differences in reproductive health and safe sex behaviors also exist in other countries. In Beijing, female students tended to have more knowledge of emergency contraception and condom use then male students [22]. About half of that sample was female (50.63%), so we might have seen the same difference if our sample had more male participants. Additionally, in Iran, female students’ knowledge about contraceptives was significantly higher than that of male students [23]. Swedish male students from three upper secondary schools were asked what topics they would like more knowledge on [24]. A quarter to a third of the male students requested more knowledge on the female reproductive system, anatomy, and functions (31%); the male reproductive health system, anatomy, and physiology (27%); and sexually transmitted infections (26%).

In our study, nine out of ten questions from the post-program quiz were statistically answered more correctly with the biggest change seen in the question regarding sperm longevity. These results were similar to a study conducted at Baylor University with 139 students. Although over 90% answered that ovulation time can vary, less than half understood the timing of conception and how it varies based on the life span of sperm [25]. A similar pilot study carried out at Jordan University evaluated an elective course [26]. Classes on reproductive health were given to students for 4 weeks as an hour-long class per day, five days a week. This class used interactive teaching methods such as brainstorming, debates, open-ended questions, group discussion, and games. One of the pre- and post-course surveys consisted of 14 reproductive health knowledge questions, and its response items were presented in a 3-point response format (yes, no, and do not know). All 14 questions showed a significant difference in the students’ knowledge between pre-course and post-course test scores.

Student pharmacists’ role in addressing reproductive health knowledge gaps has not been defined. However, the students attending seminars overwhelmingly agreed that the student pharmacists were knowledgeable about the topic, adequately addressed questions, were respectful, and remained unbiased. Pharmacists have been shown to play a key role in contraceptive access [14], so including and empowering student pharmacists early on in the process could help ensure they play supportive roles in public health upon graduation. The WHO also realizes that there is a need for the pharmacy profession to be more actively engaged in increasing access to contraception [15]. Many countries have over-the-counter contraception, which is a recent development for the U.S. with the over-the-counter O-pill (norgestrel). Some countries already allow pharmacist to prescribe contraception, such as Canada, the United Kingdom, Australia, and New Zealand [27].

The number of students sending an advocacy letter was low, which could reflect the placement of this important aspect at the end of the seminar hour, when students needed to get to class or work, go home, or were fatigued. Furthermore, students might not be skilled in political advocacy yet, or might be cautious about expressing their viewpoints.

Some limitations exist with our program evaluation. The convenience sample most likely represented students with an interest in the subject or those who were at their student organization meeting, but not necessarily because they needed the information. Students were also more likely to attend the seminar when it was offered as co-curricular credit, which suggests that students are more likely to attend when there is an additional perceived “benefit” to them. Our students were from one large urban university, although from a variety of colleges. Differences in previous sexual and reproductive health education are seen within and between states.

All quiz, program evaluation, and follow-up survey items were created by investigators without formal reliability testing. Although attendees rated the program highly, overall attendance was low, despite seminars being offered at various times of day on the main campus and on Zoom. Further research needs to be carried out to identify better recruitment strategies and a mode of learning that increases student engagement with the topics of unintended pregnancy prevention and reproductive health. Students possibly do not perceive this information as being important to them or believe an unintended pregnancy is not likely, being unaware of the statistics. According to the health belief model, students probably need more information on the susceptibility and consequences of an unintended pregnancy to increase their interest in the topic [16]. For the two-month follow-up survey, recall bias could exist with remembering details related to sexual behavior activity and their use of and adherence to medications. When comparing subgroups, no corrections were made for multiple comparisons. As the number of men and trans/nonbinary participants were low, our ability to find gender differences could have been limited by these small sample sizes. A large-scale public health education program would need to be implemented to measure actual changes in unintended pregnancy rates due to reproductive health and safe sex seminars that increase knowledge and behavior changes.

## 5. Conclusions

By providing college-aged students with a health education presentation based on the various components of the health belief model, which includes critical reproductive health information and safe sex behaviors, students can decrease their knowledge gaps on these topics and increase their self-efficacy to implement some safe sex behaviors. Potentially, this type of health education could decrease unintended pregnancies. The positive student responses to our program highlight the critical need for sexual and reproductive health programs to be available to college-aged students to fill in the gaps from grade and high school programs. Our study supports the cognitive constructivism theory and rational model that with improved knowledge, college students can not only identify health behavior attitudes and practices that need to be changed but can implement that change. Our presentation is adaptable for use in other universities and could be explored as an option to meet the needs of high school students to prevent unintended pregnancies in this age group as well. Although the program was highly valued, attendance was low, thus strategies to increase attendance at these types of programs need to be discovered. For example, universities could consider implementing short, mandatory modules covering critical reproductive health information to ensure students are receiving this information. An hour-long interactive seminar might not be the only way to deliver this information, so other modes of education should be created and evaluated. Future studies are needed to assess knowledge gaps and the best education methods, starting earlier, in grade and high school, with repeat education in college and a focus on how these can be adapted to other non-urban settings. Assessing information retention and behavior changes over longer intervals is also important. Increasing education and knowledge are helpful, but other interventions to overcome other causes of unintended pregnancies are needed.

## Figures and Tables

**Table 1:** Student attendee demographics.

Characteristic (Number Answering Item)	Results
**Age in years (140)**	
18–25	118 (84.3%)
26–31	17 (12.1%)
32–46	5 (3.6%)
**Gender (144)**	
Female	122 (84.7%)
Male	15 (10.4%)
Trans or non-binary	6 (4.2%)
Prefer not to say	1 (0.7%)
**Race (144)**	
White (European, Middle Eastern, Northern African)	96 (66.7%)
Asian or Asian American	18 (12.5%)
Black or African American	20 (13.9%)
Hawaiian or Pacific Islander	1 (0.7%)
American Indian or Alaskan Native	1 (0.7%)
More than one race	2 (1.4%)
Other	4 (2.8%)
Prefer not to say	2 (1.4%)
**Ethnicity (144)**	
Arab or Arab American	24 (16.7%)
Hispanic	9 (6.3%)
Neither	107 (74.3%)
Prefer not to say	4 (2.8%)
**Education**	
**Degree (143)**	
Certificate or Associate’s degree	4 (2.8%)
Bachelor of Science	62 (43.4%)
Bachelor of Arts	23 (16.1%)
Master of Science	8 (5.6%)
Doctor of Philosophy	39 (27.3%)
Postdoc	7 (4.9%)
**Area of study (145)**	
Arts, music or theater	5 (3.5%)
Business	9 (6.2%)
Communications	3 (2.1%)
Computers and Information Technology (IT)	3 (2.1%)
Education	7 (4.8%)
Engineering	4 (2.8%)
Health care professional	49 (33.8%)
Humanities	1 (0.7%)
Law	2 (1.4%)
Liberal arts	9 (6.2%)
Prerequisites for a health care professional degree	12 (8.3%)
Psychology	6 (4.1%)
Public health	9 (6.2%)
Science	17 (11.7%)
Sociology	2 (1.4%)
Other	7 (4.8%)

**Table 2:** Pre- and post-program quiz and program evaluation results.

Question and Correct Answer	Pre-Program Quiz Number Correct	Post-Program Quiz Number Correct	*p*-Value
**How long can sperm survive in the body, allowing for a potential pregnancy during this time? Answer**: 5–6 days	42 (29.0%)	125 (86.2%)	<0.001
**Which of the following is MOST accurate regarding fertilization within the menstrual cycle? Answer**: You can get pregnant at any point within your cycle including your period.	80 (55.2%)	132 (91.0%)	<0.001
**What are the MOST effective forms of birth control? Answer**: The implant, hormonal IUDs, and copper IUDs	71 (49.0%)	116 (80.0%)	<0.001
**How many days can you miss an oral birth control pill for the birth control to still be effective? Answer**: One day (24 h) if taken within 24 h	58 (40.0%)	119 (82.1%)	<0.001
**On average, for a hundred women who only used condoms as a contraceptive for a year, how many will get pregnant? Answer**: 13	60 (41.4%)	90 (62.1%)	<0.001
**Within how many days after unprotected sex is Plan B^®^ most effective, reducing your chances of getting pregnant by 75–89%? Answer**: Within 72 h (3 days)	87 (60.0%)	130 (89.7%)	<0.001
**Which of the following is (are) true about emergency contraceptive effectiveness? Answer**: All of the above are true. Pregnancy rates increased from 1% to 6% in women > 165 lbs who took Plan B^®^, women > 187 lbs who took the prescription emergency contraception Ella^®^ had twice the odds of failure rates compared to women who weighed < 187 lbs, and studies showed no benefit of taking double the dose of Plan B^®^ if you weigh > 165 lbs.	85 (58.6%)	87 (60.0%)	0.785
**Which of the following is true regarding male condoms? Answer:** Latex condoms must be used with water-based lubricants (not oil) to prevent breakage.	73 (50.3%)	136 (93.8%)	<0.001
**A bill is currently presented to the Michigan House of Representatives that would allow pharmacists to prescribe contraception to patients, thereby increasing its access and availability. Answer:** True	131 (90.3%)	142 (97.9%)	0.005
**Where can you currently get prescription birth control? Answer:** Your obstetrician/gynecologist, primary care provider, online telehealth platforms such as Nurx ^®^ and or CVS community pharmacies.	119 (82.1%)	140 (96.6%)	<0.001
**Will you be supporting pharmacist-prescribed contraception to your legislator?**			ND
I sent an email during the seminar.	5 (3.5%)		
I plan to send an email.	122 (86.5%)		
I don’t plan to send an email.	14 (9.9%)		
**How important do you feel continuing to offer #PlanA seminars are to the WSU student body?**			ND
Very important	131 (92.3%)		
Moderately important	10 (7.0%)		
Not important	1 (0.7%)		

IUDs: intrauterine devices, ND: not done.

**Table 3:** Students’ self-assessment and program evaluation.

Items ^1^	Strongly Agree/Agree Responses	Neutral/Disagree Responses
My knowledge about reproductive health and safe sex behaviors greatly increased as a function of attending this seminar.	140 (97.9%)	3 (2.1%)
The active learning and contraception products enhanced my understanding of contraception products and medications	135 (95.7%)	6 (4.3%)
I will change some of my sex behaviors to be safer as a function of the information learned today. [If not sexually active, please answer not applicable.] ^2^	93 (72.1%)	15 (11.6%)
The information presented today will help me prevent an unintended pregnancy.	133 (96.5%)	4 (2.9%)
Being able to obtain contraception from a pharmacist would help decrease unintended pregnancies.	131 (96.5%)	6 (4.4%)
The presentation was fair and unbiased.	133 (97.1%)	4 (2.9%)
The speakers were knowledgeable about the content.	136 (98.6%)	2 (1.4%)
Speakers adequately addressed all the questions.	131 (96.3%)	5 (3.7%)
Speakers were respectful of attendees’ viewpoints.	130 (96.3%)	5 (3.7%)
I would recommend #PlanA seminars to other students.	131 (97.0%)	4 (3.0%)

^1^ Number of respondents per item varied from 129 to 143; number of missed responses varied from 2 to 16 per item. ^2^ Excludes those answering nonapplicable.

**Table 4:** Follow-up survey responses.

General Items	Strongly Agree/Agree Responses	Neutral/Disagree Responses	Not Applicable/No Responses
The #PlanA seminar was very helpful to me to prevent future unintended pregnancies.	96 (98.0%)	2 (2.0%)	0 (0.0%)
After attending the seminar, I am more confident in making the correct birth control and reproductive health decisions for myself to prevent unintended pregnancies.	97 (99.0%)	1 (1.0%)	0 (0.0%)
After attending the seminar, I am more confident in incorporating safe sex behaviors into my relationships.	96 (98%)	2 (2.0%)	0 (0.0%)
After attending the seminar, I am more informed about the availability and correct use of emergency contraception.	96 (98.0%)	2 (2.0%)	0 (0.0%)
**Prescription Contraception User Items**	**Yes/True** **Responses**	**No/False** **Responses**	**Not Applicable/No Responses**
Are you currently taking a prescription birth control product?	42 (42.9%)	56 (57.1%)	0 (0.0%)
I have switched or made an appointment to switch the type of birth control I use (e.g., went from pills to implant). ^1^	6 (14.2%)	27 (64.3%)	9 (21.4%)
I have had a pharmacist from CVS prescribe me my birth control. ^1^	2 (4.8%)	30 (71.4%)	10 (23.8%)
I have been taking my current birth control prescription more regularly than before. ^1^	22 (52.4%)	6 (14.3%)	14 (33.3%)
I now take my current birth control pills at the same time each day. ^1^	25 (59.5%)	1 (2.4%)	16 (38.1%)
I missed one dose of my birth control and took it within 24 h. ^1^	17 (40.5%)	5 (11.9%)	20 (47.6%)
I missed more than one dose of my birth control and used emergency contraception. ^1^	5 (11.9%)	14 (33.3%)	23 (54.8%)
After missing two or more birth control pills, I used backup contraception like condoms for 7 days. ^1^	8 (19.0%)	7 (16.7%)	27 (64.3%)
**Sexually Active Items**	**Yes/Agree** **Responses**	**No/Neutral/Disagree** **Responses**	**Not Applicable/No Responses**
Have you been sexually active with a partner in the last two months?	49 (50.0%)	49 (50.0%)	0 (0.0%)
My use of condoms to prevent an unintended pregnancy has increased in response to my learning from the #PlanA seminar. ^2^	33 (67.3%)	13 (26.5%)	3 (6.1%)
As a function of the #PlanA seminar, I have changed my sexual behaviors to become more safe to prevent unintended pregnancies. ^2^	37 (75.5%)	12 (24.5%)	0 (0.0%)
How often have you had unprotected intercourse in the past two months? ^2,3^	19 (42.2%)	0 times	5 (10.2%)
	20 (44.4%)	1–5 times	
	3 (6.7%)	6–10 times	
	2 (4.4%)	11–20 times	
How many times have you had to use emergency contraception during the past two months? ^2^	43 (89.6%)	0 times	0 (0.0%)
	3 (6.3%)	1 time	
	1 (2.1%)	2 times	
	1 (2.1%)	3 times	
Is your use of emergency contraception over the last two months higher or lower than before the #PlanA seminar?	5 (10.2%)	Higher	0 (0.0%)
	35 (71.4%)	The same	
	9 (18.4%)	Lower	
**Advocacy Item**			**No Responses**
Have you emailed your Michigan House of Representatives legislator a letter to support pharmacists prescribing birth control?	7 (17.3%)	Yes.	1 (1.0%)
	24 (24.5%)	No, I will do so now.	
	39 (39.8%)	No, I will do it later.	
	17 (17.3%)	No, I don’t plan on it.	

CVS: large community pharmacy chain in the United States. ^1^ Subgroup reporting yes to prescription contraception use; *n* = 42. Nonapplicable and no responses could be related to using nonoral prescription contraception or perfect adherence. ^2^ Subgroup who were sexually active in the last two months; *n* = 49. ^3^ Excludes the outlier who answered unprotected sex 65 times in two months.

**Table 5:** Pre- and post-program survey subgroup analyses.

	No.	%	Pre-Program Score	Post-Program Score	Learning Score Change	Pre Q1-Q10 ^1^	Post Q1-Q12 ^1^	Age ^2^ *p*-Value	Gender ^2^ *p*-Value	Race ^2^ *p*-Value	Health Care Program ^2^ *p*-Value	Degree ^2^ *p*-Value
**Age**												
18–22 years old	100	71.4	5.8	8.5	2.7	Q2	Q2, Q4,		0.003	0.030	0.576	0.013
23–46 years old	40	28.6	5.1	8.1	3.0		Q7		<0.001 ^4^	0.157	0.070	0.026 ^5^
*p*-value	0.047	0.140	0.419									
**Gender ^3^**												
Female	122	89.1	5.7	8.4	2.8	Q6	Q6	0.111		0.922	0.247	0.140
Male	15	10.9	4.5	7.9	3.5			<0.001 ^4^		0.966	0.623	0.034 ^6^
*p*-value			0.022	0.220	0.202							
**Race** White Non-White												
	96	66.2	5.8	8.7	2.9		Q2, Q3,	0.159	0.75		0.090	0.284
	49	33.8	5.1	7.8	2.7		Q7, Q10	0.157	0.966		0.039 ^7^	0.254
*p*-value			0.036	<0.001	0.668							
**Health Care Program** Yes No												
	49	33.8	5.4	8.6	3.1			0.013	0.257	0.160		0.010
	96	66.2	5.6	8.3	2.7			0.010	0.623	0.039 ^7^		0.054
*p*-value			0.453	0.341	0.184							
**Degree** Undergraduate Graduate/postdoc												
	89	62.2	5.5	8.3	2.8			0.135	0.159	0.132	0.022	
	54	37.8	5.6	8.5	2.9			0.026 ^5^	0.34 ^6^	0.254	0.054	
*p*-value			0.800	0.430	0.750							

^1^ Only the numbers of the questions that are statistically significant are listed. ^2^ The numbers represent the two elements of each category. ^3^ Transgender (*n* = 2), nonbinary (*n* = 4), prefer not to say (*n* = 1), and no answer (*n* = 1) choices were excluded. ^4^ Men are more likely to be older students and women younger students. ^5^ Undergraduates are younger and graduate/postdocs are older. ^6^ Undergraduates are more often women and graduate/postdocs are more often men. ^7^ Health care students are more White and non-health care students are more non-White.

## Data Availability

All data are presented in the manuscript.
